# Dual Mechanisms of pH-Dependent Tea Astringency: Binding and Metabolism of Polyphenols Revealed by In Vivo and In Vitro Oral Processing

**DOI:** 10.3390/foods15132315

**Published:** 2026-06-29

**Authors:** Xiaoduo Ma, Jinglin Yu, Jiaoyue Zhang, Huanlu Song, Yanbo Wang, Tianyang Guo

**Affiliations:** School of Food and Health, Beijing Technology and Business University, Beijing 100048, China; mxd200104012@163.com (X.M.); 13810878791@163.com (J.Y.); 15190213929@163.com (J.Z.); songhl@th.btbu.edu.cn (H.S.); wyb1225@163.com (Y.W.)

**Keywords:** tea astringency, pH, binding, metabolism, oral processing

## Abstract

pH critically influences the astringency of tea, yet the underlying mechanisms remain poorly understood. This study aimed to elucidate how pH modulates tea astringency through dual mechanisms (polyphenol–saliva binding and polyphenol oral metabolism) with comparable *in vivo* and *in vitro* oral processing (OP) models. After optimizing OP parameters (10 mL, 10 s), the effect of pH (3.0–6.0) on green tea infusion and representative polyphenols (epigallocatechin-3-O-gallate (EGCG), epigallocatechin (EGC), and gallic acid (GA)) was investigated by sensory evaluation, turbidimetry, and paper spray mass spectrometry (PS-MS). Astringency intensity was shown to increase as pH decreased from 6.0 to 3.5, with a slight decline at pH 3.0. Turbidimetry revealed that lower pH led to greater turbidity increases after OP, indicating enhanced polyphenol–saliva binding. PS-MS revealed that lower pH was associated with greater reductions in polyphenol concentrations and increased formation of metabolites, with changes consistent with the conversion of EGCG to EGC and GA. These pH-dependent trends were consistent between *in vivo* and *in vitro* OP and were validated with single polyphenol solutions. Collectively, these findings reveal that pH modulates tea astringency by simultaneously enhancing both binding and metabolism of polyphenols. The comparable *in vivo* and *in vitro* OP approach provides a robust platform for mechanistic studies of astringency in complex food systems.

## 1. Introduction

Astringency is a distinctive oral sensation arising from the interaction between polyphenols in foods (e.g., tea) and the oral cavity [1,2,3]. This sensation is a key determinant of consumer acceptability and is fundamentally linked to the concept of oral processing (OP) [4,5]. Among the factors that modulate astringency, pH plays a particularly important role. In tea, pH varies considerably: unfermented green tea typically exhibits higher pH, while fully fermented black tea has lower pH; additionally, many flavored tea beverages are intentionally acidified. Such pH variations profoundly alter astringency perception, with most studies reporting that lower pH intensifies astringency [6,7]. However, the precise mechanisms by which pH exerts this effect remain incompletely understood.

The classical explanation for astringency involves the binding of polyphenols with salivary proteins (e.g., mucin- and proline-rich proteins), leading to precipitation and loss of oral lubrication [8,9,10]. More recently, evidence has emerged that polyphenols can also undergo oral metabolism, mediated by salivary enzymes or oral microbiota [5,11,12]. These two processes (i.e., binding and metabolism) have largely been studied in isolation, and it remains unclear how pH simultaneously influences both. The electrostatic nature of these interactions, which depends on the isoelectric points of salivary proteins and the ionization state of polyphenols, is likely a key modulator [13,14,15]. Recent molecular dynamics simulations have revealed that the binding affinity of polyphenols to proteins is pH-dependent, with stronger hydrophobic interactions at near-neutral pH and reduced binding at very low pH [16].

Several methodological gaps further hinder a comprehensive understanding. First, direct comparability between *in vivo* and *in vitro* OP is rarely established. Many studies rely on either *in vivo* sensory panels or *in vitro* model systems (e.g., mixing polyphenols with collected saliva) [5,17]. However, parameter settings vary widely, making it difficult to translate *in vitro* findings to real oral conditions or to validate *in vivo* observations under controlled laboratory settings. Second, there is a disconnect between whole food and single compounds. Some studies investigate single PC solutions to simplify the system [9,18,19,20], while others focus on complex food extracts (e.g., green tea flavanol extract or wine extracts) [21,22]. Systematic comparison between whole tea infusion and representative single polyphenols under identical OP conditions is lacking, yet such comparison is essential to determine whether mechanisms observed in simplified systems hold true in complex food matrices, where multiple polyphenols may interact with each other [23,24].

Additionally, traditional analytical methods have limitations in characterizing these processes simultaneously. Turbidimetry can effectively indicate PC–saliva binding through turbidity increase [25,26,27], but it lacks molecular specificity and therefore cannot distinguish whether the reduction in polyphenols is due to binding or oral metabolism (e.g., hydrolysis of ester-type catechins). Conversely, conventional metabolomic approaches can provide molecular information but often require extensive sample preparation [28], which is challenging for rapidly changing OP samples. A method capable of simultaneously assessing binding and oral metabolism with minimal sample handling is therefore needed. Paper spray mass spectrometry (PS-MS) offers unique advantages for this application: it is tolerant to complex matrices, requires minimal sample preparation, and enables rapid analysis [29,30,31], which is critical for capturing dynamic OP processes. In our recent work, PS-MS has been successfully applied to monitor oral metabolic changes in astringent compounds [11], revealing the dynamic changes in metabolites after oral processing.

To address these gaps, this study introduces an integrated analytical strategy that combines turbidimetry and PS-MS to simultaneously monitor polyphenol binding and metabolism during OP. Using comparable *in vivo* and *in vitro* OP models with optimized parameters (sample volume, exposure time), we systematically investigated the effect of pH (3.0–6.0) on astringency in both green tea infusion and representative single polyphenols (EGCG, EGC, GA). This design allows us to: (1) elucidate how pH modulates binding and metabolism as dual mechanisms; (2) establish direct comparability between *in vivo* and *in vitro* OP; and (3) cross-validate findings between complex food systems and simplified model solutions. By integrating these three dimensions, this study provides a comprehensive mechanistic understanding of pH-dependent tea astringency and offers a versatile platform for studying similar flavor attributes in other foods.

## 2. Experimental Section

### 2.1. Reagents and Materials

Food-grade epigallocatechin-3-O-gallate (EGCG), epigallocatechin (EGC), and gallic acid (GA) were purchased from Jinkangtai (Xi’an, China), and AR-grade EGCG, EGC, and GA were purchased from Yuanye (Shanghai, China). Food-grade malic acid, citric acid, tartaric acid, and lactic acid were purchased from Huiquan Biotechnology (Zhengzhou, China) and Jindan Lactic Acid Technology (Dancheng, China). Ethyl gallate as the internal standard (IS) was purchased from Shanghai Zzstandard (Shanghai, China). Food-grade tannin acid was purchased from Fuchen (Tianjin, China). HPLC-grade methanol and AR-grade sodium hydroxide were purchased from Mreda (Beijing, China). Ultrapure water was prepared by the ultrapure water machine (Zhongyangyongkang, Beijing, China).

### 2.2. Sample Preparation

Taiping Houkui (Yuanchun Tea Company, Huangshan, China) was selected as the representative green tea sample based on its high content and diverse profile of polyphenols as reported in our previous work [2,11], and was used in all assays described in this paper. The 3.0 g green tea sample was placed in a 150 mL sensory evaluation cup and infused with boiling water for 4 min, following the National Standard of Tea Evaluation [32]. The tea infusion was then separated from the leaves, rapidly cooled to room temperature, and its initial pH was measured using a pH meter (Mettler Toledo, Shanghai, China). The pH was adjusted to 6.0, 5.5, 5.0, 4.5, 4.0, 3.5, and 3.0 using malic acid aqueous solution.

The reference compounds (EGCG, EGC, and GA) were dissolved in water at their respective astringency threshold concentrations (190 μmol/L, 520 μmol/L, and 292 μmol/L) [1,33] and at one-fifth of the threshold concentrations (38 μmol/L, 104 μmol/L, and 58.4 μmol/L). AR-grade reference compounds were used for PS-MS detection, while food-grade reference compounds were used for sensory evaluation and turbidimetry assays. The pH of all six reference solutions was initially measured and then adjusted to 6.0, 4.5, and 3.0 with malic acid aqueous solution.

### 2.3. Sensory Evaluation

Sensory evaluation was conducted following the Sensory analysis—Evaluation guideline of astringency intensity of beverages/alcoholic beverages [34]. Tannic acid (TA) aqueous solutions at concentrations of 0.0, 200.0, 350.0, 650.0, 800.0, and 1000.0 μg/mL were used as reference standards corresponding to astringency intensities of 0, 2, 4, 6, 8, and 10 (from weak to strong), respectively.

Ten panelists (5 males and 5 females, aged 20–30 years, non-smokers, non-drinkers, with normal taste sensitivity) participated in the study. All panelists provided written informed consent, and the study protocol was approved by the Ethics Committee of Beijing Technology and Business University (No. 202622, Beijing, China). Panelists underwent training for four weeks (two 1 h sessions per week) to recognize the astringency intensities of TA reference solutions. During training, panelists were randomly presented with TA solutions and asked to rate the astringency intensity. Panelists who gave incorrect ratings received additional training until they could correctly identify all reference solutions. Training effectiveness was confirmed after each panelist could correctly evaluate the intensity of a randomly presented TA solution.

After training, panelists evaluated the astringency intensity of all tea infusions and PC solutions at different pH levels. All samples were pre-warmed to 37 °C before evaluation. Each panelist took 10 mL of sample into the oral cavity, swirled it for 10 s, expectorated, and immediately rated the astringency intensity. The oral cavity was rinsed with water after each evaluation, and a break was taken until the astringency sensation fully subsided (typically 1–2 min). Samples were coded with three-digit random numbers and presented in randomized order across panelists. Each sample was evaluated once by each panelist. Although ten panelists meet the minimum requirement of the applied standard [34] for astringency evaluation, a larger panel would further improve statistical power.

### 2.4. In Vivo Oral Processing

The healthy volunteers described in Section 2.3 were recruited for *in vivo* oral processing (OP) assays. All volunteers provided written informed consent, and the protocol was approved by the Ethics Committee of Beijing Technology and Business University (No. 202622, Beijing, China). Volunteers fasted for at least 10 h prior to the experiment and rinsed their oral cavity with water before sampling.

Samples (tea infusions and PC solutions at different pHs) were pre-warmed to 37 °C. During *in vivo* OP, each volunteer took a specified volume of sample (the “before oral processing” sample, bOP) into the oral cavity, swirled it for a defined oral processing time, and then expectorated the remaining sample (the “after oral processing” sample collected from *in vivo* processing, *in vivo* aOP) into a collection tube placed in an ice bath for rapid cooling. A series of sample volumes (5, 10, and 20 mL) and oral processing times (2, 5, 10, 20, 30, and 60 s) were tested to determine the optimal conditions. All samples were stored in an ice bath and analyzed within 8 h.

### 2.5. In Vitro Oral Processing

For *in vitro* OP assays, saliva was collected from the volunteer pool described in Section 2.4. Saliva was collected between 9:00 a.m. and 12:00 p.m. after at least 10 h of fasting. Volunteers rinsed their oral cavity with water, and the first saliva produced was discarded. Unstimulated saliva was then collected by dropper. Saliva samples were pooled (equal volumes per collection), rapidly cooled in an ice bath, and used within 8 h.

For *in vitro* OP, samples and pooled saliva were pre-warmed to 37 °C. The bOP sample was mixed with saliva at a certain ratio, and the mixture was swirled using a vortex oscillator for a certain duration (the *in vitro* oral processing time) to obtain the *in vitro* aOP (the “after oral processing” sample generated by *in vitro* simulation), which was then immediately cooled in an ice bath. The ratio and duration were optimized based on the *in vivo* OP results described in Section 3.1. All samples were stored in an ice bath and analyzed within 8 h.

### 2.6. Paper Spray Mass Spectrometry (PS-MS)

Samples (in triplicate) analyzed by PS-MS included tea infusions and PC solutions before and after *in vivo*/*in vitro* OP, as well as control samples listed in Table 1. All solutions were adjusted to pH 7.0 with sodium hydroxide aqueous solution, and IS (ethyl gallate) was added to a final concentration of 0.005 mg/mL. Then, 5.0 μL of the final solution was spotted onto a triangular chromatography paper (12 × 18 mm, Whatman 1ET, GE, Little Chalfont, UK) and dried under vacuum at 25 °C for 5 min using a vacuum dryer (Tianlin Hengtai Technology, Beijing, China). The custom-built paper spray (PS) ion source consisted of a copper clip (Yufa, Dongguan, China) fixed on an insulating holder, connected to a high-voltage generator (Dalian Dingtong, Dalian, China). The electrospray ionization (ESI) source of the mass spectrometer (6410 triple quadrupole MS, Agilent, Santa Clara, CA, USA) was replaced with the PS source. Then, 40 μL of methanol was pipetted onto the paper to desorb and ionize the analytes.

PS-MS parameters were as follows: nebulizer gas pressure, 30 psi; capillary temperature, 325 °C; drying gas flow rate, 5 L/min; internal capillary voltage, 0 V; external voltage, −4000 V; polarity, negative; mass range, *m*/*z* 50–1000. Data were processed using Agilent MassHunter Qualitative Analysis Software (B.07.00, Santa Clara, CA, USA). Compound identification was performed by comparison with reference standards, as described in our recent work [2]. Semi-quantification was achieved by calculating the peak area ratio of each analyte to the IS.

### 2.7. Turbidimetry

Turbidity was measured using a turbidimeter (WZS-186, Leici, Shanghai, China) calibrated with 20, 100, and 200 NTU standard solutions. All samples (bOP, *in vivo* aOP, and *in vitro* aOP) were gently vortexed for 10 s immediately before measurement to ensure homogeneity. Each measurement was performed at 25 °C with 20 mL of sample in a standard turbidity vial. Because the turbidimeter required a minimum volume of 20 mL, aOP samples were prepared by pooling two replicate aOP samples obtained from two independent oral processing sessions by the same volunteer (for *in vivo*) or from two identical *in vitro* reactions (for *in vitro*). For each pH condition, the pooled sample was measured within 5 min after oral processing to minimize sedimentation of insoluble complexes. Each sample was measured in triplicate, and the average turbidity value (NTU) was reported. Control experiments (water at different pH mixed with saliva) were performed under identical conditions to confirm that turbidity increases were specifically due to tea–saliva interactions rather than pH alone. The same sample set as in Section 2.6 was analyzed.

### 2.8. pH Measurement

The same sample set as in Section 2.6 was analyzed. pH values were measured using a pH meter calibrated with standard buffers at pH 4.01, 7.00, and 9.21.

### 2.9. Statistical Analysis

All instrumental measurements were performed in triplicate, and the results are expressed as mean ± standard deviation (SD). For sensory evaluation, each of the ten panelists scored each sample once; the astringency intensity of each sample is reported as the mean ± SD of the ten scores. Differences among multiple groups were evaluated using one-way analysis of variance (ANOVA) followed by Tukey’s post hoc test. Prior to ANOVA, normality was assessed by the Shapiro–Wilk test and homogeneity of variances was confirmed by Levene’s test. For comparisons identified as significant by Tukey’s HSD test, 95% confidence intervals for mean differences were computed by the standard error from the ANOVA.

## 3. Results and Discussion

### 3.1. Optimization of Experimental Parameters

To establish comparable conditions for pH adjustment and subsequent OP assays, the selection of a suitable pH adjuster was first evaluated. Four common non-volatile organic acids (malic acid, citric acid, tartaric acid, and lactic acid) were considered. Citric acid interfered with the detection of quinic acid (*m*/*z* 191.1) by PS-MS, while tartaric acid exhibited a slight astringent taste that could interfere with sensory evaluation. Lactic acid, at the high concentrations required for low pH, also affected the signal intensity of quinic acid. In contrast, malic acid showed no detectable interference in either PS-MS detection or sensory evaluation, and was therefore selected as the pH adjuster.

Following this, the key OP parameters were optimized. Three initial volumes (5, 10, and 20 mL) were tested across oral processing times (2, 5, 10, 20, 30, and 60 s), and the final expectorated volume was measured for each combination (Table 2). For all three initial volumes, the final volume at short processing times (2–20 s) was slightly lower than the initial volume, while at longer times (30–60 s) it exceeded the initial volume. This suggests a dynamic process: some tea infusion may be retained on oral surfaces initially, with saliva gradually secreted as time extends. The optimized *in vivo* OP parameters were 10 mL and 10 s (where the expectorated volume most closely matched the initial volume). Correspondingly, the *in vitro* OP conditions were set with a sample-to-saliva volume ratio of 10:1 (10 mL tea infusion mixed with 1 mL pooled saliva) and a processing time of 10 s, as determined by turbidity matching to achieve comparability in terms of overall turbidity trends. The *in vitro* system was designed to match *in vivo* parameters for pH-dependent trends but does not fully replicate oral dynamics. Thus, the observed comparability is specific to pH-dependent effects.

These parameters fall within ranges reported in previous studies, though variation exists. *In vivo* sample volumes range from 1 mL [27] to 18 mL [17], with 10 mL common [5]. *In vitro* sample-to-saliva ratios vary widely (1:2 [27] and 4:1 [22]), and a 1:1 ratio has been suggested to maximize turbidity [35]. However, our *in vivo* measurements indicated the actual ratio in the oral cavity is closer to 10:1 (10 mL sample vs. approximately 1 mL saliva secreted over 10 s). We therefore adopted a ratio that reproduces this *in vivo* condition. This approach addresses parameter variability [5,17] and provides a standardized framework for comparative analysis.

### 3.2. Sensory Evaluation of Green Tea

**Figure 1:**
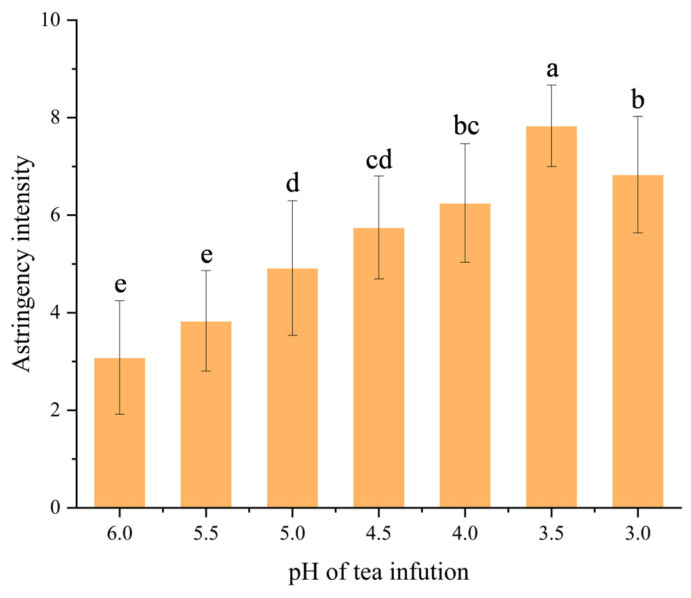
Astringency intensity evaluation results (*n* = 10) for the tea infusion with different pHs from 6.0 to 3.0 (Lowercase letters represent the significance within group (*p* < 0.05)).

With the optimized parameters (10 mL, 10 s), ten panelists evaluated the astringency intensity of tea infusions at seven pH levels (6.0 to 3.0). As shown in Figure 1, astringency intensity increased from 3.08 to 7.83 as pH decreased from 6.0 to 3.5, with a slight decrease at pH 3.0. This trend aligns with previous reports that lower pH enhances astringency [6,7]. The slight decrease at pH 3.0 could be partially explained by sourness masking, as very low pH intensifies sourness which may interfere with astringency perception [7]. Alternatively, the binding affinity between polyphenols and salivary proteins may reach a maximum near pH 3.5 and decline at more extreme pH [13,16]. Because sourness and astringency were not quantitatively separated, the observed decrease at pH 3.0 should be further investigated in future studies employing time-intensity scaling or astringency-specific reference standards.

The 10 mL sample volume matches that employed in previous astringency studies [6], where panelists expectorated after 5 s [6]. The 10 s duration selected here was based on the optimization results (Section 3.1), allowing direct comparison with chemical analyses.

### 3.3. Oral Binding of Green Tea Characterized by Turbidimetry

**Figure 2:**
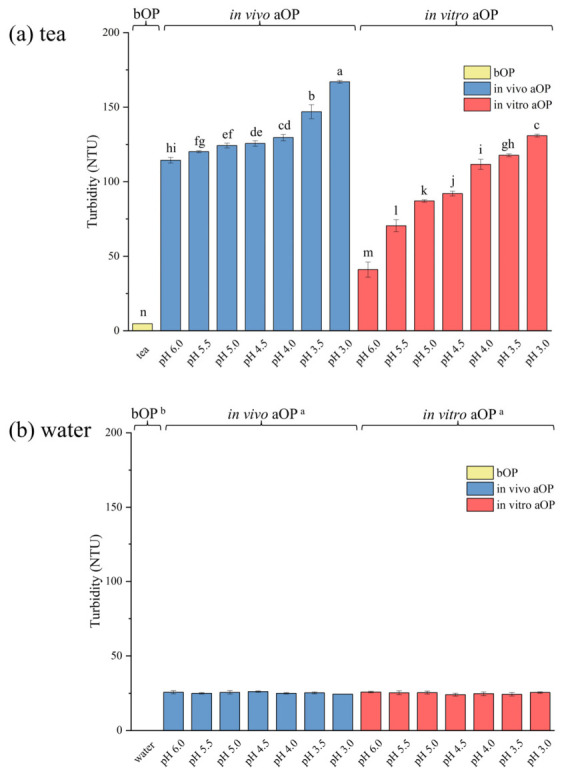
Turbidimetry results (*n* = 3) for (**a**) tea infusion and (**b**) water with different pHs from 6.0 to 3.0 reacted with the saliva during *in vivo* and *in vitro* oral processing (Lowercase letters represent the significance within group (*p* < 0.05)).

Turbidity was measured to evaluate binding between tea components and saliva. Before OP, tea infusion showed low turbidity across all pH levels (ca. 5 NTU). After OP, turbidity increased substantially: *in vivo* from 114 NTU at pH 6.0 to 167 NTU at pH 3.0 and *in vitro* from 41 NTU at pH 6.0 to 130 NTU at pH 3.0 (Figure 2a). Visually, the mixtures became progressively more turbid as pH decreased: at pH 3.0, the tea–saliva mixture appeared distinctly cloudy and opaque, whereas at pH 6.0 it remained relatively clear. This visual gradation was consistent with the measured NTU values. To exclude the possibility that pH itself directly affected turbidity, control samples were adjusted to pH 7.0 before measurement; no significant differences were observed compared to unadjusted samples (data not shown). In addition, water samples at different pH levels reacted with saliva (control group 2) showed nearly constant turbidity values (ca. 25 NTU) that were substantially lower than those of tea infusion (Figure 2b). These results indicate that the observed turbidity increase after OP is specifically due to interactions between tea components and saliva, and that lower pH enhances this interaction.

The pH-dependent binding observed here aligns with classic studies [13] and recent mechanistic findings. In a study with β-lactoglobulin (β-LG) as a model protein, the binding affinity for EGCG was shown to be highest at neutral pH and lowest at pH 2.5, with the interaction driven primarily by hydrophobic forces [16]. In our study, turbidity increased as pH decreased from 6.0 to 3.5, consistent with the trend toward higher affinity near pH 4 (the isoelectric point of many salivary proteins [14]) and the general principle that protein–polyphenol binding is favored when net charges are minimized [13]. The slight decrease at pH 3.0 may reflect a transition to less favorable binding, analogous to the reduced affinity observed for β-LG at pH 2.5 [16]. Moreover, pH influences the ionization of both polyphenols and salivary proteins and reduces the negative surface charge of salivary proteins, thereby reducing electrostatic repulsion and allowing hydrophobic interactions to dominate [14]. It has also been shown that green tea polyphenols, particularly EGCG, alter salivary mucin properties [9], and polyphenol–PRP interactions depend on galloylation and degree of polymerization [10]. The consistency between *in vivo* and *in vitro* OP further supports the reliability of our *in vitro* model for studying binding mechanisms.

### 3.4. Oral Metabolism of Green Tea Characterized by PS-MS

Before formal detection, the internal standard (IS) was evaluated for stability during OP in control group 2, and bOP samples at different pHs (adjusted to pH 7.0) in control group 1 showed consistent signal intensity. These controls confirmed the reliability of the subsequent PS-MS analysis.

**Figure 3:**
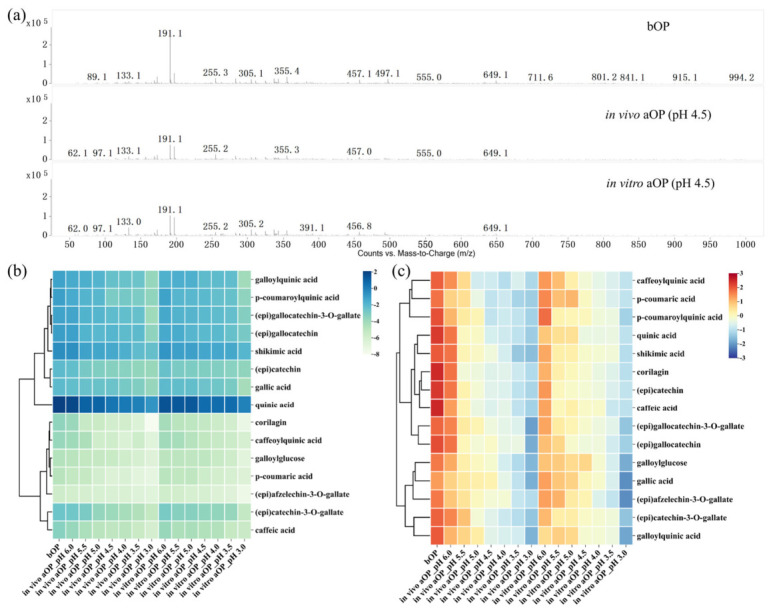
Paper spray mass spectrometry results (*n* = 3) for the tea infusion with different pHs from 6.0 to 3.0 reacted with the saliva during *in vivo* and *in vitro* oral processing (OP); (**a**) Full scan mass spectrum (subtracting background spectrum) of different states of tea infusion (bOP, *in vivo* aOP, and *in vitro* aOP); Semiquantitative results of phenolic compounds in different states of tea infusion (**b**) Semiquantitative results (log_2_ scale). (**c**) Row-standardized heatmap with color bar indicating normalized intensity range (blue: low; red: high). Darker shades after OP correspond to greater reduction in polyphenols at lower pH.

Fifteen representative polyphenols with high concentrations were identified in tea infusion by PS-MS, mainly including catechins, phenolic acids and their derivatives, and hydrolysable tannins (Table 3 and Figure 3a). Semiquantitative results of bOP, *in vivo* aOP, and *in vitro* aOP at different pHs are shown in Figure 3b,c. Before OP, quinic acid had the highest intensity, followed by (epi)gallocatechin-3-O-gallate, (epi)gallocatechin, galloylquinic acid, p-coumaroylquinic acid, and shikimic acid. After OP, all polyphenols decreased, with the reduction strongly pH-dependent: modest at near-neutral pH, more pronounced at pH 4.5, and dramatic at pH 3.0, where some polyphenols were nearly undetectable. This pattern was consistent between *in vivo* and *in vitro* OP. Reduction rates varied among polyphenols; for example, at pH 3.0, (epi)gallocatechin-3-O-gallate and (epi)gallocatechin decreased by approximately 82.4% and 85.8% *in vivo* versus 72.7% and 74.8% *in vitro*, respectively, while galloylquinic acid and p-coumaroylquinic acid decreased by about 74.6% and 82.3% *in vivo* compared to 77.4% and 82.2% *in vitro*. The reduction became more pronounced as pH decreased, aligning with the sensory results (Section 3.2) where lower pH gave higher astringency intensity.

The observed pH-dependent reduction in polyphenol signals is consistent with several possible mechanisms, including enhanced binding to salivary proteins, pH-induced chemical instability (e.g., epimerization or autoxidation), ion suppression in PS-MS analysis, and potential enzymatic hydrolysis by salivary esterases. Salivary esterases have been shown to hydrolyze galloylated polyphenols such as EGCG and tannic acid [36], and their activity may be pH-dependent. Buettner [37] demonstrated that human saliva contains esterases capable of hydrolyzing esters, and the extent of hydrolysis depends on the chemical structure of the substrate. More recently, Liu et al. [36] directly showed that EGCG and tannic acid are hydrolyzed by salivary esterases, leading to significant degradation of these galloylated polyphenols. Lower pH may also increase the accessibility of ester bonds, potentially facilitating hydrolysis—whether enzymatic or non-enzymatic. The consistency between *in vivo* and *in vitro* OP indicates that the *in vitro* system reproduces the observed pH-dependent trends. This supports the utility of the *in vitro* model for studying these trends, but does not prove an exclusively enzymatic mechanism. Nevertheless, the PS-MS data are consistent with the occurrence of enzymatic hydrolysis alongside binding and other pH-dependent effects. Our previous work has shown that oral metabolism of polyphenols is dynamic, varying with time and location; residual polyphenols are highest within the first 2 min after tea consumption and decline after 10 min, with phenolic acids persisting for over 60 min [11].

### 3.5. Validation with PC Solutions by Turbidimetry

To validate the binding behavior observed in tea infusion, representative polyphenols (EGCG, EGC, and GA) were examined under identical OP conditions at two concentration levels (astringency threshold and one-fifth threshold) across three pH values (6.0, 4.5, and 3.0).

**Figure 4:**
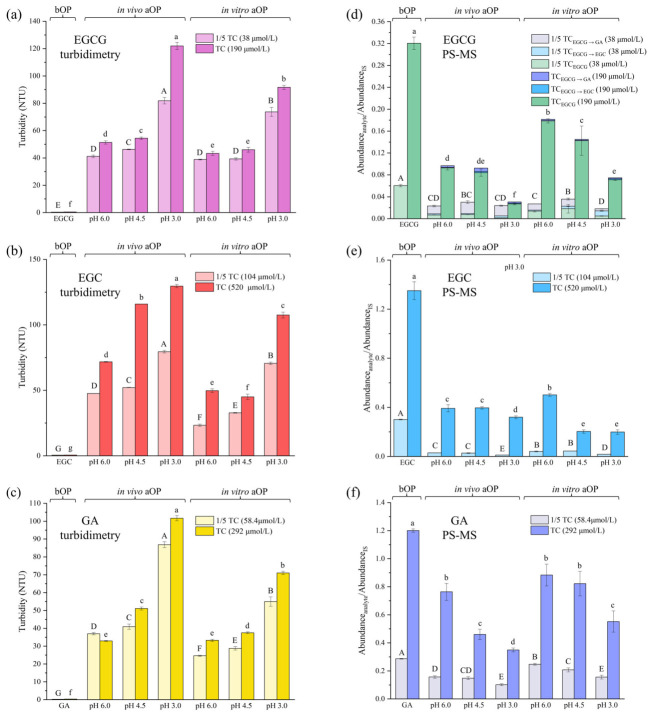
Turbidimetry results (*n* = 3) for (**a**) EGCG, (**b**) EGC, and (**c**) GA, and paper spray mass spectrometry results (*n* = 3) for (**d**) EGCG, (**e**) EGC, and (**f**) GA with three pHs at two levels (threshold concentrations and 1/5 threshold concentrations) reacted with the saliva during *in vivo* and *in vitro* oral processing (Different uppercase letters indicate significant differences at the 1/5 threshold concentration level (*p* < 0.05), while different lowercase letters indicate significant differences at the threshold concentration level (*p* < 0.05)) The arrows (EGCG → GA and EGCG → EGC) indicate that gallic acid (GA) and epigallocatechin (EGC) were detected as hydrolysis products derived from EGCG after oral processing.

As shown in Figure 4a–c, turbidity increased after OP for all three polyphenols, more pronounced at lower pH. For EGCG at threshold concentration, turbidity values were 51 NTU at pH 6.0, 54 NTU at pH 4.5, and 122 NTU at pH 3.0. Similar trends were observed for EGC and GA. Among the three, EGC showed a greater difference in turbidity between the two concentration levels; for example, at pH 3.0, turbidity increased from 71 NTU at the lower concentration to 107 NTU at threshold concentration. These results suggest that the pH-dependent binding in tea infusion reflects the behavior of individual polyphenols, which exhibit distinct concentration–response relationships. Turbidimetry measures aggregate formation as an indirect indicator of polyphenol–protein binding. Direct quantification of binding stoichiometry or affinity requires complementary techniques such as surface plasmon resonance or isothermal titration calorimetry.

The structural features of polyphenols significantly influence their binding strength. The higher turbidity induced by EGCG compared to GA is consistent with previous findings that galloylated polyphenols interact more strongly with salivary proteins [10,13]. This parallels tribological findings that tannins (higher molecular weight) induce greater friction than gallic acid [38], highlighting the role of molecular weight and galloylation in astringency mechanisms. The use of turbidity to evaluate polyphenol–protein interactions has been validated previously, with strong correlations (r > 0.9) between turbidity and astringency intensity for galloylated polyphenols [27].

Machine learning models have also demonstrated that molecular fingerprint similarities can predict astringency thresholds and types for flavonoid compounds, with galloylation and B-ring hydroxylation being key structural determinants [39,40]. Targeted metabolic profiling of green tea polyphenols further highlights the roles of hydroxylation, glycosylation, acylation, and condensation in tea astringency [41].

### 3.6. Validation with PC Solutions by PS-MS

The PC solutions described in Section 3.5 were analyzed by PS-MS to validate the metabolic changes observed in tea infusion. After OP, concentrations of EGCG, EGC, and GA decreased, with greater reductions at lower pH (Figure 4d–f). For EGCG at the threshold concentration, the degree of reduction under *in vivo* and *in vitro* conditions were as follows: 69.8% and 43.3% at pH 6.0; 71.2% and 54.8% at pH 4.5; and 90.4% and 76.7% at pH 3.0, respectively.

Notably, in EGCG solutions, the appearance of EGC and GA was detected after OP, suggesting that hydrolysis of EGCG to EGC and GA may occur during oral processing. This potential conversion was more pronounced at lower pH and higher initial concentrations. However, the PS-MS data alone cannot distinguish whether this hydrolysis is enzymatic (catalyzed by salivary esterases), non-enzymatic (acid-catalyzed ester cleavage), or partially due to preferential loss of EGCG through protein binding. Whether this hydrolysis is enzymatic or non-enzymatic cannot be determined from the present data. Nevertheless, the pH dependence of this conversion aligns with the known activity of salivary esterases, which have been shown to hydrolyze galloylated polyphenols in a pH-dependent manner [36]. These results suggest that the changes observed in tea infusion are consistent with the involvement of ester-type catechins and occur independently of the complex tea matrix.

Together, turbidimetry and PS-MS suggest that pH modulates tea astringency through two concurrent processes: enhanced polyphenol–saliva binding and increased oral metabolism. These processes are not independent; binding may reduce free polyphenols for metabolism, while metabolic products may themselves participate in binding. The consistent pH-dependent trends across both systems suggest pH shifts the equilibrium of this coupled system. Previous studies have also shown that non-galloylated catechins influence salivary protein secretion and salivary film stability [23], suggesting binding and metabolism involve dynamic equilibrium processes.

### 3.7. pH Change After Oral Processing

**Figure 5:**
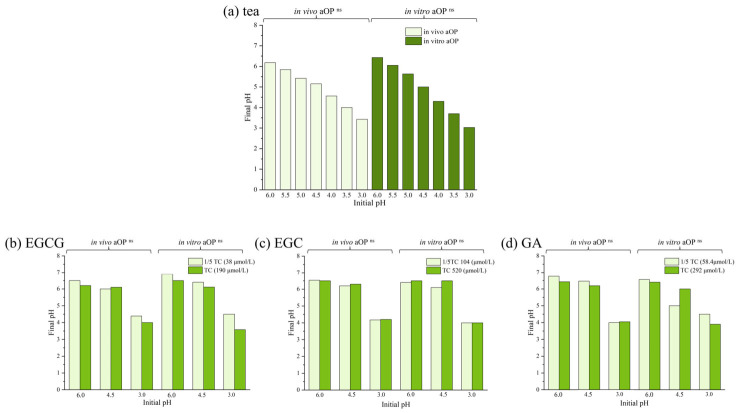
The final pH results for (**a**) tea infusions (*n* = 3) with different pHs from 6.0 to 3.0 and (**b**) EGCG, (**c**) EGC and (**d**) GA with three pHs at two levels (threshold concentrations and 1/5 threshold concentrations) reacted with the saliva during *in vivo* and *in vitro* oral processing (ns indicates no significant difference between groups).

The pH values of samples before and after OP were measured (Figure 5). The final pH of aOP samples was generally slightly higher than that of bOP samples. For tea infusion, the pattern was clear: at low initial pH (3.0–4.5), final pH *in vivo* was slightly higher than *in vitro*; at high initial pH (5.0–6.0), final pH *in vivo* was slightly lower than *in vitro*. This suggests the *in vitro* system is more sensitive to pH changes than *in vivo*. For PC solutions, pH changes were larger than for tea infusion, with less clear patterns and notable fluctuations at initial pH 4.5. These differences likely reflect the buffering capacity of saliva [17], which may also contribute to the observed variations in binding and metabolism between whole tea and single compounds.

## 4. Conclusions

This study established comparable *in vivo* and *in vitro* oral processing (OP) models with optimized parameters (10 mL, 10 s), revealing that both polyphenol–saliva binding and oral polyphenol metabolism exhibit consistent pH-dependent trends across the two systems. The consistency of these trends indicates that the *in vitro* model reproduces the observed pH-dependent trends seen *in vivo*, rather than fully replicating all physiological aspects of oral processing. Through the combined use of turbidimetry and paper spray mass spectrometry (PS-MS), pH was found to modulate tea astringency through dual mechanisms—enhanced binding (reflected by turbidity increase) and changes consistent with increased metabolism—as indicated by the detection of newly formed metabolites such as EGC and GA from EGCG. By comparing whole tea infusion with representative single polyphenols (EGCG, EGC, GA), the observed pH-dependent behaviors were found to be intrinsic to the polyphenols themselves, while the complex tea matrix modulates the overall intensity through competitive interactions. Together, these findings provide a comprehensive mechanistic understanding of pH-dependent tea astringency and offer a versatile analytical strategy applicable to other astringent foods.

Building on this established platform, future mechanistic studies can employ *in vitro* OP with precisely controlled conditions, such as temperature, incubation time, enzyme inhibition (e.g., esterase inhibitors), and protein-free controls, to dissect the relative contributions of binding and metabolism. Complementary techniques including direct binding assays (e.g., surface plasmon resonance) and molecular dynamics simulations should further elucidate how pH regulates the molecular interactions between polyphenols and oral receptors at the atomic level. Together, these approaches allow robust *in vitro* models to replace *in vivo* screening for deeper mechanistic studies of pH-dependent astringency.

## Figures and Tables

**Table 1:** Experimental design of experimental group and control groups.

	Factor A	Factor B	Factor C
	pH Regulation	Tea Infusion ^a^	Saliva ^b^
experimental group	√	√	√
control group 1	√	√	×
control group 2	√	×	√
control group 3	×	√	√
control group 4	×	×	√
control group 5	×	√	×
control group 6	√	×	×
control group 7	×	×	×

“√” indicates the presence of this factor, while “×” indicates its absence. ^a^ “×” in Factor B means the tea infusion was instead of water. ^b^ “×” in Factor C means the saliva was instead of water.

**Table 2:** Change in final volumes of tea infusion during *in vivo* oral processing at different initial volumes.

OP Time (s)	Final Volume (mL)		
	5 mL Initial Volume	10 mL Initial Volume	20 mL Initial Volume
0	5 ^bc^	10 ^bc^	20 ^b^
2	4.64 ± 0.21 ^c^	9.87 ± 0.06 ^c^	19.20 ± 0.20 ^e^
5	4.80 ± 0.17 ^cd^	9.87 ± 0.09 ^c^	19.53 ± 0.30 ^d^
10	4.83 ± 0.08 ^cd^	9.90 ± 0.08 ^c^	19.61 ± 0.10 ^d^
20	4.93 ± 0.12 ^bc^	9.95 ± 0.05 ^bc^	19.83 ± 0.15 ^c^
30	5.17 ± 0.15 ^ab^	10.17 ± 0.06 ^ab^	19.93 ± 0.21 ^bc^
60	5.35 ± 0.18 ^a^	10.33 ± 0.29 ^a^	20.37 ± 0.21 ^a^

Note: Lowercase letters represent the significance within group (*p* < 0.05).

**Table 3:** Qualitative results of phenolic compounds in tea infusion by paper spray mass spectrometry.

No	Phenolic Compounds	Abbr.	Category	Charge	*m*/*z*
1	p-coumaric acid		phenolic acids	[M-H]^-^	163.0
2	gallic acid	GA	phenolic acids	[M-H]^-^	169.0
3	shikimic acid		phenolic acid derivatives	[M-H]^-^	173.0
4	caffeic acid		phenolic acids	[M-H]^-^	179.0
5	quinic acid		phenolic acid derivatives	[M-H]^-^	191.1
6	(epi)catechin	(E)C	catechins	[M-H]^-^	289.1
7	(epi)gallocatechin	(E)GC	catechins	[M-H]^-^	305.1
8	galloylglucose		hydrolysable tannin	[M-H]^-^	331.1
9	p-coumaroylquinic acid		phenolic acid derivatives	[M-H]^-^	337.1
10	galloylquinic acid; theogallin		phenolic acid derivatives	[M-H]^-^	343.1
11	caffeoylquinic acid		phenolic acid derivatives	[M-H]^-^	353.1
12	(epi)afzelechin-3-O-gallate		catechins	[M-H]^-^	425.1
13	(epi)catechin-3-O-gallate	(E)CG	catechins	[M-H]^-^	441.1
14	(epi)gallocatechin-3-O-gallate	(E)GCG	catechins	[M-H]^-^	457.1
15	corilagin		hydrolysable tannin	[M-H]^-^	633.1

## Data Availability

The data presented in this study are available on request from the corresponding author.
